# Antibacterial Properties and Effects of Fruit Chilling and Extract Storage on Antioxidant Activity, Total Phenolic and Anthocyanin Content of Four Date Palm (*Phoenix dactylifera*) Cultivars

**DOI:** 10.3390/molecules21040419

**Published:** 2016-03-26

**Authors:** Muhammad Azizan Samad, Siti Hajar Hashim, Khanom Simarani, Jamilah Syafawati Yaacob

**Affiliations:** 1 Institute of Biological Sciences, Faculty of Science, University of Malaya, 50603 Kuala Lumpur, Malaysia; azizan_12@siswa.um.edu.my (M.A.S.); hajarhashim93@gmail.com (S.H.H.); hanom_ss@um.edu.my (K.S.); 2 Centre for Research in Biotechnology for Agriculture (CEBAR), Institute of Biological Sciences, Faculty of Science, University of Malaya, 50603 Kuala Lumpur, Malaysia

**Keywords:** antioxidant activity, total phenolic content, total anthocyanin content, storage, antibacterial activity, *Phoenix dactylifera*

## Abstract

*Phoenix dactylifera* or date palm fruits are reported to contain natural compounds that exhibit antioxidant and antibacterial properties. This research aimed to study the effect of fruit chilling at 4 °C for 8 weeks, extract storage at −20 °C for 5 weeks, and extraction solvents (methanol or acetone) on total phenolic content (TPC), antioxidant activity and antibacterial properties of Saudi Arabian *P. dactylifera* cv Mabroom, Safawi and Ajwa, as well as Iranian *P. dactylifera* cv Mariami. The storage stability of total anthocyanin content (TAC) was also evaluated, before and after storing the extracts at −20 °C and 4 °C respectively, for 5 weeks. Mariami had the highest TAC (3.18 ± 1.40 mg cyd 3-glu/100 g DW) while Mabroom had the lowest TAC (0.54 ± 0.15 mg cyd 3-glu/100 g DW). The TAC of all extracts increased after storage. The chilling of date palm fruits for 8 weeks prior to solvent extraction elevated the TPC of all date fruit extracts, except for methanolic extracts of Mabroom and Mariami. All IC_50_ values of all cultivars decreased after the fruit chilling treatment. Methanol was a better solvent compared to acetone for the extraction of phenolic compounds in dates. The TPC of all cultivars extracts decreased after 5 weeks of extract storage. IC_50_ values of all cultivars extracts increased after extract storage except for the methanolic extracts of Safawi and Ajwa. Different cultivars exhibited different antibacterial properties. Only the methanolic extract of Ajwa exhibited antibacterial activity against all four bacteria tested: *Staphylococcus aureus*, *Bacillus cereus*, *Serratia marcescens* and *Escherichia coli*. These results could be useful to the nutraceutical and pharmaceutical industries in the development of natural compound-based products.

## 1. Introduction

*Phoenix dactylifera* or date palm is a monocotyledon, dioecious plant in the Palmaceae family which inludes about 210 to 220 genera and 1500 to 2500 species [1]. The Food and Agricultural Organization of the United Nations (FAO) estimated that in 2012 Egypt was the largest date palm producer, followed by Iran, Saudi Arabia, Algeria, Iraq, Pakistan, Oman, United Arab Emirates, Tunisia and Libya [2].

Date palm fruits had been assessed for the presence of antioxidant compounds such as anthocyanins, vitamins, carotenoids and phenolic compounds [3,4]. Antibacterial properties were also exhibited by date palm extract, which could inhibit the growth of Gram positive and Gram negative bacteria [5,6].

Over the decades there has been an increase in awareness and knowledge among consumers regarding the health benefits of antioxidants. Furthermore, continuous research for antibacterial compounds is a race against the emergence of antibiotic resistant bacteria [7]. These two major issues drive much of the research done on plants to discover their antioxidant compounds and antibacterial properties. Plants are often targeted for this purpose because they are naturally immobilized at a place, which forces them to be adaptable to biotic and abiotic stressors. Bacterial infection is one type of biotic stress that a plant has to overcome. In order to fight against this biotic stress, plants develop resistance mechanisms, which typically involves the synthesis of different metabolites [8]. 

Therefore, this research was conducted to evaluate the presence of antioxidant compounds and antioxidant activity as well as antibacterial properties in four *P. dactylifera* cultivars, namely, Mabroom, Safawi, Ajwa and Mariami, which may have the potential to be developed as natural compound-based products by the nutraceutical and pharmaceutical industries.

## 2. Results and Discussions

### 2.1. Effect of Solvent Type on Extraction Efficiency

**Figure 1:**
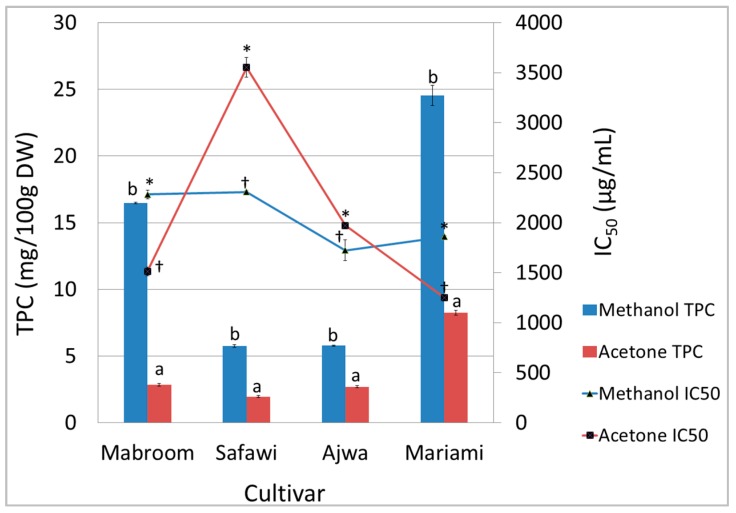
Effect of extraction solvent on TPC (mg GAE/100 g DW) and IC_50_ (μg/mL) of different *P. dactylifera* cultivars. Values are the mean ± standard error of a minimum of three samples. The mean with different letters or symbols differs significantly at *p* < 0.05 (subject to different cultivars).

Methanol and acetone have the same polarity index, *i.e.*, 5.1 [9]. Both solvents were used to examine the extraction of polar compounds, specifically tannins. Date palm fruits are rich in tannins which are reported to be extracted better with absolute methanol [4]. It was also reported that the addition of water to methanol in the extraction of date palm components reduced the extraction efficiency [10]. Therefore, absolute acetone was used as a comparison due its similar polarity index. It was discovered that all methanolic extracts had higher TPC compared to acetone extracts (Figure 1). The mass of extracts yielded from the methanol extraction method (67% to 92% of 1.0 g) was higher compared to the acetone extraction method (12% to 31% of 1.0 g), nevertheless all the IC_50_ values of the acetone extracts were lower than those of the methanolic extracts, except for the Safawi and Ajwa samples, suggesting that the acetone extracts had more DPPH radical scavenging activity compared to the methanolic extracts.

Methanol has higher viscosity and is relatively more polar than acetone, even though they have the same polarity index [9]. The solvent polarity and the solubility of compound to be extracted are two factors that affect the extraction process [11]. Based on the results, it was revealed that methanol could extract the phenolic compounds from date palm fruits better than acetone. These results are supported by previous findings which showed that acetone did not perform well in phenolic compound extraction compared to methanol [12,13]. The efficiency of the phenolic compound extraction by methanol and acetone might also be different depending on the plant species [14]. Other studies showed that methanol was also efficient in extracting certain compounds such as catechin, epicatechin and epigallocatechin [15,16]. Even if absolute methanol and acetone could extract the same phenolic compounds, the efficiency of extraction of different phenolic compounds could vary [17], thus affecting the TPC as a whole. It was reported in previous works that acetone could also extract antioxidant enzymes such as peroxidase and catalase [18,19] while methanol could extract polyphenol oxidase which could oxidize phenolic compounds [20,21]. The presence of antioxidant enzymes such as catalase in the acetone extracts might also function as the scavengers of the DPPH radicals [22]. Thus, in short, the solvents used might not just extract phenolic compounds but also other compounds such as oxidative or antioxidant enzymes which could also be responsible for a decrease or increase in the antioxidant activities in the methanolic and acetone extracts of the different date palm cultivars.

### 2.2. Effect of Fruit Chilling Prior to Extraction

In this study, the effect of fruit chilling (at 4 °C for 8 weeks) prior to extraction on the total phenolic content (TPC) and DPPH scavenging activity of methanolic and acetone extracts of four different *P. dactylifera* cultivars were investigated. Based on Table 1, it could be observed that the TPC of all date fruit extracts were elevated after fruit chilling, except for the methanolic extracts of Mabroom and Mariami. The TPC of Mabroom, Safawi, Ajwa and Mariami acetone extracts increased by 180.35%, 217.86%, 56.51% and 59.64% respectively, after 8 weeks of fruit chilling. Meanwhile, the TPC of Safawi and Ajwa methanolic extracts had increased by 23.61% and 133.74%, respectively. On the other hand, the methanolic extracts of Mabroom and Mariami showed a decrease in TPC after 8 weeks of fruit chilling, with a TPC loss of 37.62% and 90.38%, respectively.

The effect of fruit chilling on DPPH radical scavenging activities of the four different *P. dactylifera* cultivars was also investigated. The results were interpreted as 50% inhibition concentration (IC_50_) in μg/mL, as shown in Table 2. Interestingly, all IC_50_ values decreased after the fruit chilling treatment, and yet, the decrement was not significantly different in the methanolic extract of Safawi. The decrease in IC_50_ values was an indication that the extract concentration required to inhibit DPPH radical scavenging activity by 50% was lowered, indicating an increase in the antioxidant capacity. Ajwa fruits extracted with acetone showed the highest decrease among all extracts after 8 weeks of chilling at 4 °C.

Our results showed lower TPC values in the four tested cultivars (Mabroom, Safawi, Ajwa and Mariami) compared to the TPC of Tunisian Allig, Deglet Nour and Bejo varieties [23]. Factors such as cultivars, geographic origins, growing conditions and soil type may affect the TPC in dates [24]. The variation of proanthocyanins and phenolic compounds may explain the difference of the phenolic content in different date palm fruit varieties [25]. Besides that, Mabroom, Safawi and Ajwa dates originate from Medina, Saudi Arabia whereas Mariami dates originate from Iran. This might also contribute to the differences in the TPC as the agricultural practices of each country may be different. The increase in the TPC after fruit chilling may be due to the activity of phenylalanine ammonia lyase which is stimulated by the ethylene produced in the fruits as a result of chilling [26,27]. It is the key enzyme in the preliminary step for the channeling of carbon from primary metabolism to phenylpropanoid secondary metabolism [28]. The elevation in the activity of phenylalanine ammonia lyase, followed by the rise in ethylene production was observed in citrus fruit subjected to chilling injury [29].

On the other hand, the decrease of the phenolic content may be due to the action of glycosidase, phenolase and polyphenol oxidase activity [18,30]. The biosynthesis of these enzymes was suggested to be stimulated by ethylene [18]. The activities of these enzymes were also dependent on the pH, temperature, salts and inhibitors [18]. In date palm, flavans and chlorogenic acids were susceptible to phenolase as ripening proceeds, which caused a decrease in insoluble tannins [31]. The activity of polyphenol oxidase might also be elevated due to chilling injury [32]. Other than enzymatic reactions, the TPC decrease could also be the result of non-enzymatic reactions such as the oxidation of ascorbic acid to dehydroascorbic acid which produces hydrogen peroxide as a by-product and which may adversely affect the phenolic compounds due to their biochemical properties as antioxidants [33]. The production of ascorbic acid and dehydroascorbic acid could increase due to chilling stress [34]. Moreover, oxygen radicals were also suggested to be induced during chilling stress [34,35]. In this study, only the methanolic extracts of Mabroom and Mariami showed a reduction in TPC after 8 weeks of fruit chilling. These may be due to the activity of polyphenol oxidase in the methanolic extracts, which became elevated due to the chilling injury. 

Based on the results, all IC_50_ values observed in this study were lower compared to that reported by Kchaou *et al.* [36], which ranged from 16,700 to 46,790 μg/mL, depending on the varieties. The antioxidant activity of all extracts had been elevated and most apparent elevation was observed in the acetone extract of Ajwa. This result was in agreement with Kondo *et al*., where blueberries stored at low temperature for 3 months showed an increment in antioxidant activity [37]. Similarly, the antioxidant capacity of banana skin also increased during storage [38]. The TPC of the extracts before and after fruit chilling was also plotted against the reciprocal of IC_50_ (1/IC_50_), and showed poor linear correlations (R^2^ = 0.0028 and 0.0449, before and after chilling treatment, respectively). This indicates that the antioxidant activity was not solely dependent on the amount of TPC but also on the level of polymerization and hydroxylation of the phenolic compounds [39,40]. It is known that phenolic compounds were not the sole constituent of the extracts. Other compounds such as enzymes could also be present. It was mentioned that peroxidase could be extracted by using acetone [18], and its presence is stimulated during chilling stress. It was reported that other antioxidant enzymes such as superoxide dismutase, guaiacol peroxidase and ascorbate peroxidase also had elevated activity during chilling stress [34]. Furthermore, in this study, during the Folin-Ciocalteau’s assay for phenolic content, the samples were incubated at room temperature, therefore causing elevation of temperature in the samples. According to Shen *et al.* [34], levels of antioxidant compounds such as ascorbic acid and reduced glutathione increase after rewarming occurs. The temperature elevation in the samples may contribute to an increase in antioxidant compound levels in the extracts, thus increasing the antioxidant activities, as observed in this study.

### 2.3. Effect of Extract Storage on Stability of TPC and Antioxidant Potential 

**Figure 2:**
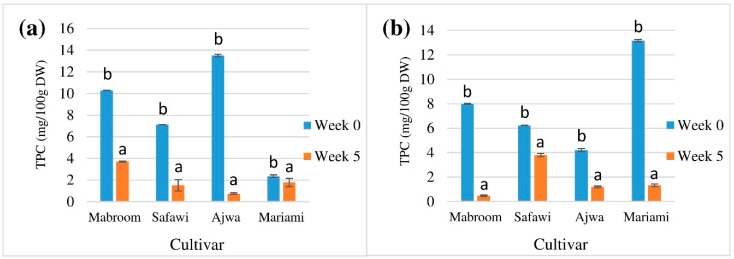
Effect of extract storage at −20 °C on TPC (mg GAE/100 g DW) of four different *P. dactylifera* cultivars, extracted using different solvents; (**a**) extraction using methanol, (**b**) extraction using acetone. Values are the mean ± standard error of a minimum of three samples. The means with different letters on a bar differed significantly at *p* < 0.05 (subject to different cultivars).

In this study, the total phenolic content in chilled fruits of four different *P. dactylifera* cultivars, extracted using methanol and acetone was compared, before and after 5 weeks of extract storage at −20 °C. Based on Figure 2, the TPC of both methanolic and acetone extracts showed significant reductions after being stored at −20 °C for 5 weeks, and the TPC of the methanolic extracts of Mabroom, Safawi, Ajwa and Mariami decreased by 64.01%, 78.79%, 94.45% and 24.58%, respectively. On the other hand, 94.12%, 39.17%, 71.50% and 89.98% decreases were observed in the TPC of acetone extracts of Mabroom, Safawi, Ajwa and Mariami respectively. Lower decrease percentages may indicate a more stable extract. Hence, among the methanolic extracts, Mariami date extract was the most stable, whereas among the acetone extracts, Safawi date extract was the most stable. 

It was revealed that the TPC of all extracts generally decreased after 5 weeks of storage, parallel to the results reported in previous studies on fruit juices [37,41,42]. In a similar study on acai fruit, which is a relative of date palm, a decrese in phenolic compounds also occurred during juice storage [43], due to decreases in procyanidin and *p*-coumaric acid concentrations, resulting in an overall polyphenolic loss. Klimczak *et al.* suggested that phenolic compounds such as free and conjugated hydroxycinnamic acids in orange juices were also affected during storage [42]. The decrease of phenolic content during frozen storage may be due to the oxidation of the antioxidant compounds [41]. Therefore it could be inferred that the decrease of TPC after extract storage observed in this study may be due to the reduction of the most abundant phenolic constituent in the extract, due to oxidation. 

There was a very high correlation between the percentage of DPPH radical scavenging activity and extract concentration, shown by R^2^ > 0.96 (data not shown). IC_50_ values of extracts of all cultivars increased after extract storage, except for the methanolic extracts of Safawi and Ajwa (Table 3). As for Mabroom and Mariami, the increase in IC_50_ values in the methanolic extracts was not significantly different (Table 3). The elevation of IC_50_ values indicates a reduction of antioxidant capacity, and *vice versa*. Methanolic extracts of Safawi and Ajwa also became more reactive against DPPH after 5 weeks of extract storage.

Based on the results, methanolic extracts were observed to be more stable compared to acetone extracts, as a lower increase in IC_50_ values was observed in methanolic extracts compared to acetone ones. The decrease in the antioxidant activity of extracts after extract storage was in agreement with previous studies [42,43]. Liolios *et al.* also reported similar observations, where higher antioxidant activity was not associated with higher amount of TPC [44]. There was a poor linear correlation between TPC and 1/IC_50_ before (R^2^ = 0.0449) and after (R^2^ = 0.0011) extract storage. The reactivity of phenols is dependent on the level of methylation, methoxylation or other group substituents since phenols act as proton donors [45]. The oxidation of ascorbic acid within the extracts might also result in the lower antioxidant activity, as previously reported [46]. The presence of stable compounds upon storage such as flavanones may also affect the stability of the extracts [42]. Other than that, different samples may contain different antioxidant compounds, thus, the behavior and reactivity of these compounds are also different. These reasoning may explain the elevation or loss of antioxidant capacity shown by the date fruit extracts.

### 2.4. Total Anthocyanin Content (TAC) and Effect of Extract Storage on Anthocyanin Stability

**Figure 3:**
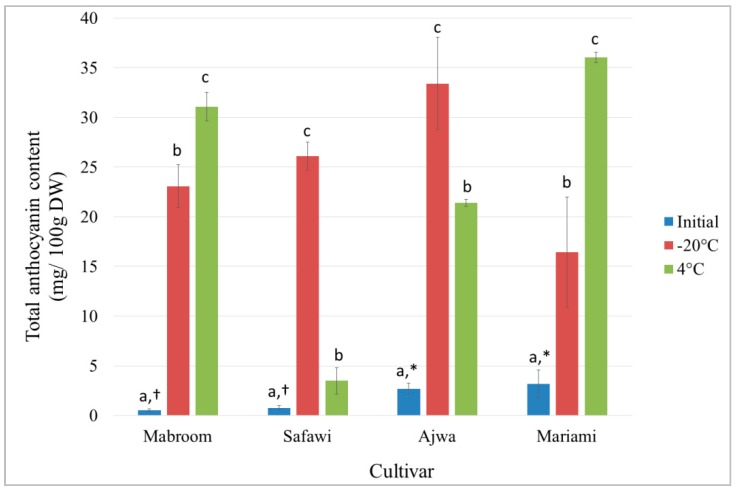
Effect of storage temperature (−20 °C and 4 °C) on the stability of anthocyanin content in different *P. dactylifera* cultivars, after 5 weeks. Values are the mean ± standard error of a minimum of three samples. The means with different letters and/or symbols differed significantly at *p* < 0.05 (subject to different cultivars).

In this study, the anthocyanin content in the fruits of four different *P. dactylifera* cultivars was also compared. As shown in Figure 3, Mariami fruits had the highest initial anthocyanin content (3.18 ± 1.40 mg cyd 3-glu/100 g DW) while Mabroom had the lowest (0.54 ± 0.15 mg cyd 3-glu/100 g DW). Excess and unused fruit extracts were also stored at −20 °C and 4 °C for 5 weeks, and compared for its anthocyanin stability. The results are shown in Figure 3. After 5 weeks of extract storage at −20 °C and 4 °C, interestingly, the anthocyanin content in all samples increased tremendously, in both treatments. The analysis of results indicated that the anthocyanin content in Mabroom and Mariami fruit extracts increased more when the extracts were stored at 4 °C compared to the storage at −20 °C, in contrast to Safawi and Ajwa (Figure 3).

Al-Farsi *et al.* discovered that the anthocyanin content in fresh Omani date palm fruits varied depending on variety and it was absent in sun-dried dates due to the damage caused by sunlight [24]. In this study, the fruits were freeze-dried instead of sun-dried to prevent the degradation of anthocyanins. Although the anthocyanin content in the extracts increased after 5 weeks of extract storage at −20 °C and 4 °C, the anthocyanin content in date palm extracts was still relatively lower compared to fruits rich in anthocyanin such as blackberries (1004.9 mg cyd 3-glu/100 g DW) [47], blueberries (580–1370 mg cyd 3-glu/100 g DW) [48], and black raspberry (540–559 mg cyd 3-glu/100 g DW) [49].

The increase in the anthocyanin content after extract storage may be associated with the decrease of extract pH after storage (Table A1). At pH 1.0 to 3.1, where anthocyanin compounds such as cyanidin 3-glucoside and petanin mainly exist in flavylium cationic form at least 90% of the colour was stable for 60 days [50]. It may also be due to the presence of colour-stable proanthocyanins which resulted from the reaction of anthocyanins with other compounds such as cinnamic acid, which is prevalent in date palm fruits [51,52]. Other than that, the tremendous increase of anthocyanins in the extract could also be due to self-association, co-pigmentation and acylation [33]. It was established that the high concentration of anthocyanins did not follow Beer’s Law [53] because of the association and stacking effect of anthocyanins at high concentration which enhances the absorbance. In other words, hypothetically, these factors masked the real amount of anthocyanin content in the extracts. At lower temperature, the chemical reactions between anthocyanins and cinnamic acids commonly are slower [54]. Nevertheless, hypothetically, the degree of association and stacking effect of anthocyanins may cause the significant observed differences between the increases of anthocyanin content between the date palm varieties after extract storage. 

### 2.5. Antibacterial Activity

Based on Table 4, none of the 100 mg/mL acetone extracts exhibited any antibacterial activity. The lowest concentration of methanolic extract of Mabroom that could inhibit *S. aureus* growth was 100 mg/mL. The lowest concentration of methanolic extract of Ajwa and Mariami that could inhibit *S. aureus* growth was 300 mg/mL. This indicated that the methanolic extract of Mabroom was more potent against *S. aureus* than methanolic extract of Ajwa and Mariami. The lowest concentration of methanolic extracts of Mabrooom and Mariami that could inhibit *B. cereus* growth was 500 mg/mL while methanolic extracts of Safawi and Ajwa could inhibit *B. cereus* growth at 400 mg/mL. There was no inhibition zone observed for *E. coli* for the methanolic extracts of Mabroom and Mariami. However, Safawi and Ajwa showed similar antibacterial activity against *E. coli*. None of the extracts exhibited antibacterial activity against *S. marcescens* except for the methanol extract of Ajwa whereby 300 mg/mL was the lowest concentration observed that could inhibit *S. marcescens* growth.

The antibacterial properties of date palm fruit have been proven by various studies [5,55,56]. Based on the results (Table 4), different cultivars possessed different antibacterial property. Ajwa exhibits antibacterial activity against all four bacteria, Mabroom and Mariami did not exhibit any antibacterial activity against Gram negative bacteria, while Safawi did not exhibit antibacterial activity against *S. marcescens*. Shakiba *et al.* reported that methanolic extracts of Mazafati cultivar could not inhibit the growth of *E. coli* PTCC 1270, *E. coli* PTCC 1399 and *S. marcescens* [57]. However, the extract was able to inhibit the growth of *E. coli* PTCC 1330 [57]. This shows that the antibacterial properties of the methanolic extract of date palm could also possibly be selective against certain strain of bacteria. Other published reports also showed that the methanolic extract of date varieties such as Mosaifah, Ruthana, Sukkari, Nabtet Ali, Medjool and Rushodia also exhibited antibacterial activity against *E. coli* [5]. As for *S.* *marcescens*, it had been discovered that *P. dactylifera* (date palm) in Kuwait was susceptible to this bacteria and it caused bacterial pink rot on the plant inflorescence [58]. This showed that the date palm could not either protect itself against *S. marcescens* or it did not produce the compounds that could fight against infection from this bacterium due to its pathogenic properties. Hence, this was probably the reason for the absence of antibacterial activity against *S. marcescens* for Mabroom, Safawi and Mariami. 

The presence of antimicrobial properties in date palm was most probably due to the presence of antioxidant compounds, phenolic compounds, tannins, alkaloids, flavonoids and steroids. All of these compounds had been proven to be able to inhibit the growth of microorganisms and fight against bacterial infection [56]. These compounds may act individually as biological active compound or provide a synergistic effect to achieve the antibacterial properties [56].

## 3. Materials and Methods

### 3.1. Plant Material

Mabroom, Safawi, Ajwa and Mariami dates (500 g) were purchased from Mercu Cita Manufacturing Sdn. Bhd. (Mahnaz Food) Shah Alam, Selangor, Malaysia (3.044290, 101.552069). Mabroom, Safawi and Ajwa dates were from Medina, Saudi Arabia while Mariami dates were from Iran. Information regarding the cultivars and origin were known based on the information supplied by the supplier. Deseeded date fruits (200 g) of were freeze-dried using a Labconco freeze dryer (Labconco, Kansas City, MO, USA) at −50 °C. The freeze-dried dates were macerated, transferred into air-tight jars wrapped with aluminium foil and stored at 4 °C. For fruit chilling treatment, the date fruits stored at 4 °C for 8 weeks were used.

### 3.2. Extraction and Quantification of Bioactive Compounds

#### 3.2.1. Determination of Total Phenolic Content (TPC) 

The extraction process was conducted as described in previous studies [59,60]. Macerated freeze-dried date fruits (1.0 g) were subjected to solvent extraction using 99.8% methanol or 99.5% acetone (30 mL). The extractions were conducted separately under dark conditions, at room temperature for seven days. Next, the solution was centrifuged at 9000 rpm at 25 °C for 5 min, from which the supernatant was collected, and then transferred into the evaporating flask of a Rotavapor^®^ R-3 (Büchi Labortechnik AG, Flawil, Switzerland) to evaporate the excess solvent. The concentration of the solvent-free extract was adjusted to 20 mg/mL using 99.8% methanol before being used in the quantification of TPC. In this study, the TPC in date fruits before and after fruit chilling at 4 °C for 8 weeks were compared. The effect of extract storage at −20 °C for 5 weeks on the TPC of the extract was also investigated.

The total phenolic content assay was conducted based on the method described in previous studies [61,62] with minor modifications. Briefly, 7.5% aqueous sodium carbonate (2.5 mL) was added into a test tube, followed by 10% Folin-Ciocalteau’s reagent (2.5 mL) and the sample (0.5 mL). The mixture was incubated in the dark for 30 min at room temperature. Blanks were also prepared by the addition of 99.8% methanol (0.5 mL) instead of the sample. As the next step, the absorbance was determined at 765 nm by using a Lambda 25 UV-Vis spectrophotometer (Perkin Elmer^™^, Waltham, MA, USA). The TPC was then determined from the standard calibration curve (R^2^ = 0.9184), using gallic acid as standard (0.001–0.006 mg/mL). The TPC in the extracts was expressed as mg gallic acid equivalent per 100 g dry weight (mg GAE/100 g DW).

#### 3.2.2. DPPH Radical Scavenging Activity

The DPPH free radicals scavenging assay was conducted to determine the antioxidant activity of the extracts as previously described [60], with minor modifications. Briefly, DPPH solution (1 mL) and diluted sample (1 mL, 10–1000 μg/mL) were mixed and shaken in a test tube. Then, 10 μg/mL extract (1 mL) was added into the test tube. The mixture was incubated for 30 min in the dark, at room temperature. The absorbance was read at 517 nm using a Lambda 25 UV-Vis spectrophotometer. The procedure was repeated on ascorbic acid samples of the same concentration (10–1000 μg/mL) as a standard comparison. The control used in this assay was DPPH (1 mL) added with 99.8% methanol (1 mL) in a test tube. The blank used in this assay was 99.8% methanol (2 mL). In this study, the DPPH scavenging activity in date fruits before and after fruit chilling at 4 °C for 8 weeks were compared. The effect of the extract storage at −20 °C for 5 weeks on the antioxidant potential of the extract was also investigated. The percentage of the DPPH radical scavenging activity was calculated using the following formula:

$$\text{DPPH radical scavenging activity}\;(\%)=\frac{A_{0}-A_{1}}{A_{0}}\times 100$$

where, A_0_ = absorbance of control, A_1_ = absorbance of sample. The graphs of percentage of DPPH radical scavenging activity against concentration were plotted by performing non-linear regression (third degree polynomial) as previously described [63]. The results were interpreted as 50% inhibition concentration (IC_50_) values expressed in μg/mL. 

#### 3.2.3. Determination of Total Anthocyanin Content (TAC)

The extraction process used for the total anthocyanin content (TAC) assay was conducted as previously described [64,65] with some modifications. In this procedure, macerated freeze-dried date fruits (10.0 g) were subjected to solvent extraction using 99.8% methanol (100 mL), conducted in the dark, at room temperature. The crude extract was centrifuged at 9000 rpm at 25 °C for 5 min using a Hettich Zentrifugen Universal 32 R (Tuttlingen, Germany). The supernatant was then transferred into clean test tubes wrapped with aluminium foil to be used in a pH-differential analysis. To evaluate the effects of extract storage on anthocyanin stability, the anthocyanin content of the methanolic extracts stored at −20 °C and 4 °C for 5 weeks was compared.

TAC in date palm fruits was determined through the pH differential method as described by previous research [66] with minor modifications. The pH of the methanolic date extract was adjusted to pH 1.0 and pH 4.5, and afterwards the absorbance of the samples was measured at 510 and 700 nm. The TAC was calculated using the following formula:

$$\text{Total anthocyanin content (mg cyd 3-glu/L)}\;=\;\frac{\text{Ab × MW × DF × 1000}\;}{\varepsilon \;\text{× L × m}}$$

where,

Ab = (A_510nm_ − A_700nm_) _pH1.0_ − (A_510nm_ − A_700nm_) _pH4.5_

Molecular weight (MW) of cyd 3-glu = 449.2 g/mol

Extraction coefficient (ε) = 29,600 mol/g

Dilution factor (DF) = 10

The results were expressed in mg cyanidin 3-glucoside/100 g dry weight (mg cyd 3-glu/100 g DW).

### 3.3. Determination of Antibacterial Activity

The antibacterial properties in date palm fruits were evaluated using the diffusion disc method [67]. Precultures of *Staphylococcus aureus*, *Bacillus cereus*, *Escherichia coli* and *Serratia marcescens* were spread on the surface of nutrient agar (NA) using a sterile cotton swab. Sample (10 μL) was dropped onto the sterile diffusion disc and then transferred onto the prepared Petri plate containing lawned bacteria. Sterile distilled water was used as negative control. Positive controls used were 100 mg/mL ampicillin and 30 μg/disc tetracycline disc for Gram positive and Gram negative bacteria, respectively. All plates were then incubated in the dark, at 37 °C for 24 h. Antibacterial activity *i.e*., the formation of halo (inhibition) zone and the diameter of inhibition zones were measured.

### 3.4. Statistical Analysis

All values were the means of at least three samples. Data were presented as mean ± standard error. Duncan’s test, *t*-test and one-way analysis of variance (ANOVA) were used to compare the results with α = 5% (SPSS Statistics version 22 for Windows, IBM Inc., Armonk, NY, USA).

## 4. Conclusions

Based on the data analysis, it could be concluded that storage of all four date palm fruit extracts at 4 °C and −20 °C increased the anthocyanin content. The chilling of date palm fruits for 8 weeks prior to solvent extraction increased the TPC of all date fruit extracts except for the methanolic extracts of Mabroom and Mariami cultivars. The antioxidant activity of all four date palm fruit cultivars also increased after fruit chilling treatment. This could be practiced in everyday life to obtain the maximum benefits from date fruits. Different cultivars also exhibited different antibacterial properties.

## Figures and Tables

**Table 1:** Effects of fruit chilling at 4 °C prior to extraction on the total phenolic content, TPC (mg GAE/100 g DW) in fruits of four different *P. dactylifera* cultivars were extracted using different solvents.

Cultivar	Solvent	TPC (mg GAE/ 100 g DW)	
		Week 0	Week 8
Mabroom	Methanol	16.48 ± 0.07 ^b^	10.28 ± 0.04 ^a^
	Acetone	2.85 ± 0.11 ^a^	7.99 ± 0.36 ^b^
Safawi	Methanol	5.76 ± 0.10 ^a^	7.12 ± 0.02 ^b^
	Acetone	1.96 ± 0.07 ^a^	6.23 ± 0.00 ^b^
Ajwa	Methanol	5.78 ± 0.03 ^a^	13.51 ± 0.12 ^b^
	Acetone	2.69 ± 0.08 ^a^	4.21 ± 0.02 ^b^
Mariami	Methanol	24.53 ± 0.74 ^b^	2.36 ± 0.11 ^a^
	Acetone	8.25 ± 0.20 ^a^	13.17 ± 0.36 ^b^

Values are the mean ± standard error of a minimum of three samples. The mean with different letters in a row differed significantly at *p* < 0.05.

**Table 2:** Effect of fruit chilling at 4 °C prior to extraction on IC_50_ (μg/mL) in fruits of four *P. dactylifera* cultivars extracted using different solvents.

Cultivar	Solvent	IC_50_ (μg/mL)	
		Week 0	Week 8
Mabroom	Methanol	2282.88 ± 43.19 ^c^	1379.24 ± 40.75 ^b^
	Acetone	1512.68 ± 42.76 ^c^	1323.17 ± 74.63 ^b^
Safawi	Methanol	2306.90 ± 12.07 ^b^	2179.67 ± 157.36 ^b^
	Acetone	3552.33 ± 98.38 ^c^	451.67 ± 11.67 ^b^
Ajwa	Methanol	1722.09 ± 103.75 ^c^	1377.33 ± 66.74 ^b^
	Acetone	1970.97 ± 2.65 ^c^	53.80 ± 1.31 ^a^
Mariami	Methanol	1860.76 ± 9.79 ^c^	1188.95 ± 2.79 ^b^
	Acetone	1250.79 ± 17.06 ^c^	130.77 ± 2.62 ^b^

Values are the mean ± standard error of a minimum of three samples. The mean with different letters in a row differed significantly at *p* < 0.05.

**Table 3:** Effect of chilled fruits extract storage at −20 °C on IC_50_ (μg/mL) of different *P. dactylifera* extracted using different solvents.

Cultivar	Solvent	IC_50_ (μg/mL)	
		Week 0	Week 5
Mabroom	Methanol	1379.24 ± 40.75 ^b^	1412.74 ± 47.35 ^b^
	Acetone	1323.17 ± 74.63 ^b^	2476.12 ± 1.00 ^c^
Safawi	Methanol	2179.67 ± 157.36 ^c^	152.66 ± 3.53 ^b^
	Acetone	451.67 ± 11.67 ^b^	577.20 ± 51.95 ^c^
Ajwa	Methanol	1377.33 ± 66.74 ^c^	1121.67 ± 27.98 ^b^
	Acetone	53.80 ± 1.31 ^a^	1385.50 ± 8.89 ^c^
Mariami	Methanol	1188.95 ± 2.79 ^b^	1190.28 ± 56.00 ^b^
	Acetone	130.77 ± 2.62 ^b^	2280.08 ± 5.33 ^c^

Values are the mean ± standard error of a minimum of three samples. The means with different letters in a row differed significantly at *p* < 0.05.

**Table 4:** Effect of different treatments on the diameter (mm) of the inhibition zone of different bacteria.

Gram	Bacteria	Treatment	Diameter of Inhibition Zone (mm)			
			Mabroom	Safawi	Ajwa	Mariami
**Positive**	*S. aureus*	dH_2_O	ND	ND	ND	ND
		Amp	15.8 ± 0.3 ^d^	6.3 ± 0.3 ^a^	6.5 ± 0.3 ^c^	16.3 ± 0.9 ^c^
		A	0	0	0	0
		Ma	0.3 ± 0.3 ^a^	0	0	0
		Mb	3.3 ± 1.7 ^b^	0	0	0
		Mc	4.2 ± 0.8 ^b^	0	1.0 ± 0.3 ^a^	2.7 ± 2.7 ^a^
		Md	10.0 ± 0.6 ^c^	0	2.0 ± 0.7 ^a^	9.5 ± 0.3 ^b^
		Me	15.3 ± 0.3 ^d^	0	4.0 ± 1.5 ^b^	17.5 ± 0.3 ^c^
	*B. cereus*	dH_2_O	ND	ND	ND	ND
		Amp	7.3 ± 0.3 ^a^	9.0 ± 0.5 ^b^	6.8 ± 0.6 ^b^	6.7 ± 0.3 ^a^
		A	0	0	0	0
		Ma	0	0	0	0
		Mb	0	0	0	0
		Mc	0	0	0	0
		Md	0	1.3 ± 0.6 ^a^	1.0 ± 0.3 ^a^	0
		Me	13.5 ± 1.5 ^b^	1.3 ± 0.6 ^a^	2.0 ± 0.7 ^a^	5.0 ± 5.0 ^a^
**Negative**	*E. coli*	dH_2_O	ND	ND	ND	ND
		Tet	11.0 ± 1.7	5.7 ± 0.7 ^b^	7.3 ± 0.3 ^b^	10.7 ± 1.2
		A	0	0	0	0
		Ma	0	0	0	0
		Mb	0	0	0	0
		Mc	0	0	0	0
		Md	0	1.0 ± 0.0 ^a^	1.0 ± 0.3 ^a^	0
		Me	0	1.0 ± 0.3 ^a^	1.0 ± 0.3 ^a^	0
	*S. marcescens*	dH_2_O	ND	ND	ND	ND
		Tet	10.0 ± 0.0	8.3 ± 0.9 ^a^	12.7 ± 1.5 ^b^	15.7 ± 9.1
		A	0	0	0	0
		Ma	0	0	0	0
		Mb	0	0	0	0
		Mc	0	0	3.0 ± 0.0 ^a^	0
		Md	0	0	5.5 ± 0.3 ^a^	0

Values are the mean ± standard error of a minimum of three samples. The mean with different letters in a column differed significantly at *p* < 0.05 (subject to different bacteria). dH_2_O: distilled water, Amp: 100 mg/mL ampicillin, Tet: 30 μg/disc tetracycline, A: 100 mg/mL acetone extract, Ma: 100 mg/mL methanolic extract, Mb: 200 mg/mL methanolic extract, Mc: 300 mg/mL methanolic extract, Md: 400 mg/mL methanolic extract, Me: 500 mg/mL methanolic extract, ND: not detected.

## Appendix A

**Table A1:** The pH of different *P. dactylifera* cultivars methanolic extracts after 5 weeks of storage at different temperatures.

Treatment (Storage Temperature)	pH			
	Mabroom	Safawi	Ajwa	Mariami
Initial	6.40	6.32	6.38	6.46
−20 °C	5.86	5.75	5.83	5.96
4 °C	1.69	1.76	1.59	1.62
